# Towards harmonization of microscopy methods for malaria clinical research studies

**DOI:** 10.1186/s12936-020-03352-z

**Published:** 2020-09-04

**Authors:** Mehul Dhorda, El Hadji Ba, J. Kevin Baird, John Barnwell, David Bell, Jane Y. Carter, Arjen Dondorp, Lenny Ekawati, Michelle Gatton, Iveth González, Philippe J. Guérin, Sandra Incardona, Ken Lilley, Didier Menard, François Nosten, Peter Obare, Bernhards Ogutu, Piero L. Olliaro, Ric N. Price, Stéphane Proux, Andrew R. Ramsay, John C. Reeder, Kamolrat Silamut, Cheikh Sokhna, Research Malaria Microscopy Working Group

**Affiliations:** 1 WorldWide Antimalarial Resistance Network, 60th Anniversary Chalermprakhiat Building 3rd Floor, 420/6 Ratchawithi Road, Ratchathewi, Bangkok, 10400 Thailand; 2 Centre for Tropical Medicine and Global Health, Nuffield Department of Medicine, University of Oxford, Oxford, UK; 3 Mahidol-Oxford Tropical Medicine Research Unit, Faculty of Tropical Medicine, Mahidol University, Bangkok, Thailand; 4 UMR 257 IRD VITROME, Campus IRD-UCAD, Dakar, Senegal; 5 Eijkman-Oxford Clinical Research Unit, Jakarta, Indonesia; 6 US Centers for Disease Control and Prevention, Atlanta, USA; 7 Independent consultant, Issaquah, WA USA; 8 Amref Health Africa Headquarters, Nairobi, Kenya; 9 Queensland University of Technology, Brisbane, Australia; 10 Terre des Hommes Foundation, Geneva, Switzerland; 11 WorldWide Antimalarial Resistance Network, Oxford, UK; 12 FIND, Geneva, Switzerland; 13 Australian Defence Force Malaria and Infectious Disease Institute, Enoggera, Australia; 14 Institut Pasteur du Cambodge, Phnom Penh, Cambodia; 15 Shoklo Malaria Research Unit, Mae Sot, Thailand; 16 Kenya Medical Research Institute, Kisumu, Kenya; 17 Global Health Division, Menzies School of Health Research and Charles Darwin University, Darwin, NT Australia; 18 School of Medicine, University of St Andrews, St Andrews, UK; 19 Special Programme for Research and Training in Tropical Diseases (TDR), a Co-sponsored Programme of UNICEF, UNDP, the World Bank and WHO, Geneva, Switzerland

**Keywords:** Malaria, Microscopy, Harmonization, Clinical research, Diagnostic, Standard

## Abstract

Microscopy performed on stained films of peripheral blood for detection, identification and quantification of malaria parasites is an essential reference standard for clinical trials of drugs, vaccines and diagnostic tests for malaria. The value of data from such research is greatly enhanced if this reference standard is consistent across time and geography. Adherence to common standards and practices is a prerequisite to achieve this. The rationale for proposed research standards and procedures for the preparation, staining and microscopic examination of blood films for malaria parasites is presented here with the aim of improving the consistency and reliability of malaria microscopy performed in such studies. These standards constitute the core of a quality management system for clinical research studies employing microscopy as a reference standard. They can be used as the basis for the design of training and proficiency testing programmes as well as for procedures and quality assurance of malaria microscopy in clinical research.

## Background

Microscopy continues to play an important role in malaria diagnosis and in research studies. While the advent of rapid diagnostic tests have reduced its importance as a primary diagnostic test in routine practice in some countries, microscopy remains an essential tool to support clinical research, severe malaria case management and monitoring of anti-malarial treatment efficacy [1]. In the specific context of drug efficacy trials, assessments of in-use and new drugs depend on high-quality microscopy to differentiate *Plasmodium* species and stages, and estimate parasite density [2]. Microscopy is likely to remain relevant in drug efficacy monitoring as initial signs of resistance to anti-malarial drugs are commonly seen first through a reduction in parasite clearance rate [3, 4]. A particular case is that of resistance to artemisinins, since the only currently available method for its detection in vivo is the measurement of parasite clearance rates, a procedure that requires repeated accurate, precise, and standardized estimations of parasite density in peripheral blood. Furthermore, malaria vaccine trials commonly use malaria parasite detection by microscopy as the endpoint [5], though this is increasingly being superseded by tests based on nucleic acid amplification. Finally, microscopy continues to be used as a reference standard for evaluation of other diagnostic tools [6]. In the absence of alternative validated field-applicable methods for malaria parasite detection, identification and quantification, microscopy will continue to be a mainstay of clinical and operational research in malaria in the medium to long term.

Standardization of methods enables direct comparisons of results from studies conducted at varying points in time and location while improving the quality and reliability of results generated in individual studies. This is particularly relevant to preparing and staining slides and for the detection, identification and quantification of malaria parasites in thick and thin blood films given their importance in determining the endpoints and outcomes of malaria trials. Various factors beyond the quality of the blood film and the reading process can also influence microscopy results. These include stochastic variation such as the distribution of parasites throughout a film, the workload and expertise of readers, how slides are declared negative or parasite density is estimated, and how discrepant results are handled, all of which can influence outcomes, particularly relating to parasite quantitation and detection of low-density infections.

Guidelines and procedures for quality assurance in malaria microscopy have already been published and are an excellent basis for standardizing and improving clinical malaria microscopy [7–10]. However, the standards which form the basis of the recommendations in these documents do not address the different, and in some respects more stringent, requirements of malaria microscopy for research. Whilst malaria diagnosis primarily needs to ensure that a patient with fever caused by malaria parasites receives appropriate anti-malarial therapy, research requirements demand more stringent standards in assessing and confirming technical performance. Research applications usually necessitate identification or exclusion of parasitaemia (as distinct from identification of malarial disease) with very high positive predictive value and accurate quantitation of parasites. The costs of false positive results may be relatively light in routine clinical settings as they would likely result in some unnecessary treatment of patients with anti-malarial drugs. However, incorrectly identifying new or continuing infection has major implications for determining the effectiveness of malaria vaccines and therapeutics.

The minimum quality assurance standards for research malaria microscopy proposed in this article would enable better comparison of results between studies conducted by various institutions. The standards have been reviewed by a committee of experts under the auspices of the Special Programme for Research and Training in Tropical Diseases (TDR) at the World Health Organization (WHO) and are the basis of the Research Malaria Microscopy Standards (ReMMS) manual [11]. An associated tool, the ‘Obare Method Calculator’, enables ready incorporation of these methods into research studies. It is envisaged that adherence to such standards will greatly enhance the value of malaria research and surveillance, and hence better guide the evolution of malaria programmes.

### Study personnel

The generation of accurate, precise and reproducible microscopy data can only be accomplished from correctly prepared, stained and examined blood films, which in turn depend on the inputs of skilled, well-trained and motivated laboratory personnel. Attaining and maintaining high standards in the preparation and staining of blood films should be routine given the availability of correct tools, standard operating procedures, and materials (see later sections of this article) but the examination of slides requires a different approach. A research microscopist is expected to be able to detect, identify and count human malaria parasites in Giemsa-stained peripheral blood films while reliably differentiating parasites from other features normally found in the film and from artefacts or debris that may be indistinguishable from parasites to the inexperienced eye. Extensive training and experience are required to attain the necessary level of proficiency, but it is hard to standardize their content and duration such that this level of proficiency can be assured. Work practices, workload and the working environment can further affect microscopists’ performance. Minimum competency standards to be attained in formal testing as a pre-requisite to performing microscopy for research studies can however be defined with reference to the particular requirements of research microscopy.

Standardized assessments for competency in malaria microscopy are currently based on a slide set described in the WHO Malaria Microscopy Quality Assurance Manual [8]. This slide set and related proficiency standards were designed to meet the needs of quality assurance in national malaria control programmes wherein parasite detection and quantification of *Plasmodium falciparum* malaria parasites are of prime importance to ensure patients with malaria receive the most appropriate anti-malarial treatment. The ‘competence level’ of microscopists is defined based on competence in detecting parasitaemia, species identification and parasite quantification, with no specific criteria defined for diagnostic sensitivity or specificity. As also indicated in the WHO Malaria Microscopy Quality Assurance Manual, specialized microscopy in clinical trials would require a more thorough assessment with an expanded slide set to obtain more robust estimates of sensitivity, specificity and other diagnostic performance criteria for each microscopist being tested.

The proposed slide set for assessing the competency of research microscopists comprises 130 slides (Box 1), and competence levels determined from assessments using this set are listed in Table 1. The most important change with respect to existing clinical microscopy guidelines follows from the critical importance of high specificity in research microscopy, given the disproportionate effect of a positive microscopy result during patient follow-up on the estimated efficacy of an anti-malarial drug or vaccine. This is reflected in the introduction of a false positive rate criterion with stringent thresholds at each accreditation level (≤ 2.5% for false positives vs. ≤ 10% for false negatives at Level 1) and in the four-fold higher number of negative slides (80 vs. 20 in the set described in the Manual). The higher number of slides allows more robust estimates of sensitivity (Se) and specificity (Sp) (errors of < 10% if Se at 90% and < 5% if Sp at 97.5%) and the inclusion of poor-quality slides to test proficiency in slide quality assessment. The minimum requirements for numbers and types of slides defined in this set could be increased to improve the precision of the assessment metrics, but this needs to be balanced against practicality given that a test with the slide set recommended here could take up to three days. However, given the size of investments in drug and vaccine development, this investment in assuring the quality of clinical trials should be seen as critical.

**Table 1:** Grades for certification of research malaria microscopists [11]

Competency level	Detection of parasitaemia	Species identification	Parasite quantification (± 25% of true count)	False positive rate
Level 1	≥ 90%	≥ 90%	≥ 50%	≤ 2.5%
Level 2	80 to < 90%	80 to < 90%	40 to < 50%	≤ 5%
Level 3	70 to < 80%	70 to < 80%	30 to < 40%	≤ 10%
Level 4	< 70%	< 70%	< 30%	> 10%

Demonstrated competency of malaria microscopists is one of several components of the process leading to high-quality research microscopy. The quality of equipment and materials along with systematic quality control (QC) and feedback directly influence the performance of microscopists (see later sections) as does the volume of work. Microscopists’ performance and workloads need to be carefully monitored and managed to ensure that operator fatigue does not adversely affect the quality of microscopy. A research microscopist may be expected to read approximately 40 slides per day depending on the proportion of positive slides and on the parasite density on those slides. More precise estimates of ideal workloads have been calculated elsewhere, but would need to be adapted to take into account the expected numbers of positive slides at patient recruitment and on follow-up according to the research protocol as well as the increased number of fields to be read before declaring a slide negative [8].

BOX 1: Recommended slide set for Competence Assessment of Research Microscopists

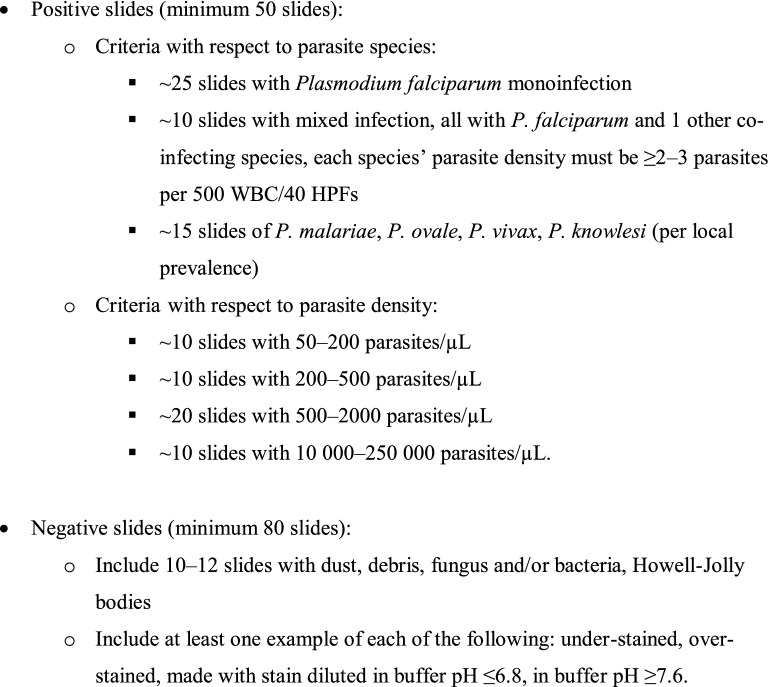

### Equipment and materials

Detailed lists of equipment and materials required for the preparation, staining and examination of slides have been published in various malaria microscopy guides and manuals [8, 9]. These have been supplemented with suggested specifications where these are not already defined in existing manuals to facilitate consistent and standardized performance of research microscopy (Box 2). The use of Giemsa (stain) and buffers from reputable manufacturers, purchased as ready-made concentrates or in powder or tablet form, would help to reduce variation in slide staining quality. Similarly, using synthetic immersion oils which are colour-stable, non-corrosive and of controlled refractive index would help maintain the performance of the microscope and the quality of the microscopic images. Some specifications, such as the recommendation for using microscopes with ocular lenses of Field Number (FN) 20 and plan-achromat objectives, are those of microscopes commonly used in malaria diagnosis and have been used as the basis for procedural details for slide examination. Care must be taken to maintain the quality of equipment and materials after procurement—this implies storage of materials and equipment in appropriate conditions, use of materials before expiry dates if applicable, and in the case of microscopes, ensuring routine care and preventive maintenance is performed as described in previously published guides [9].

**Fig. 1:**
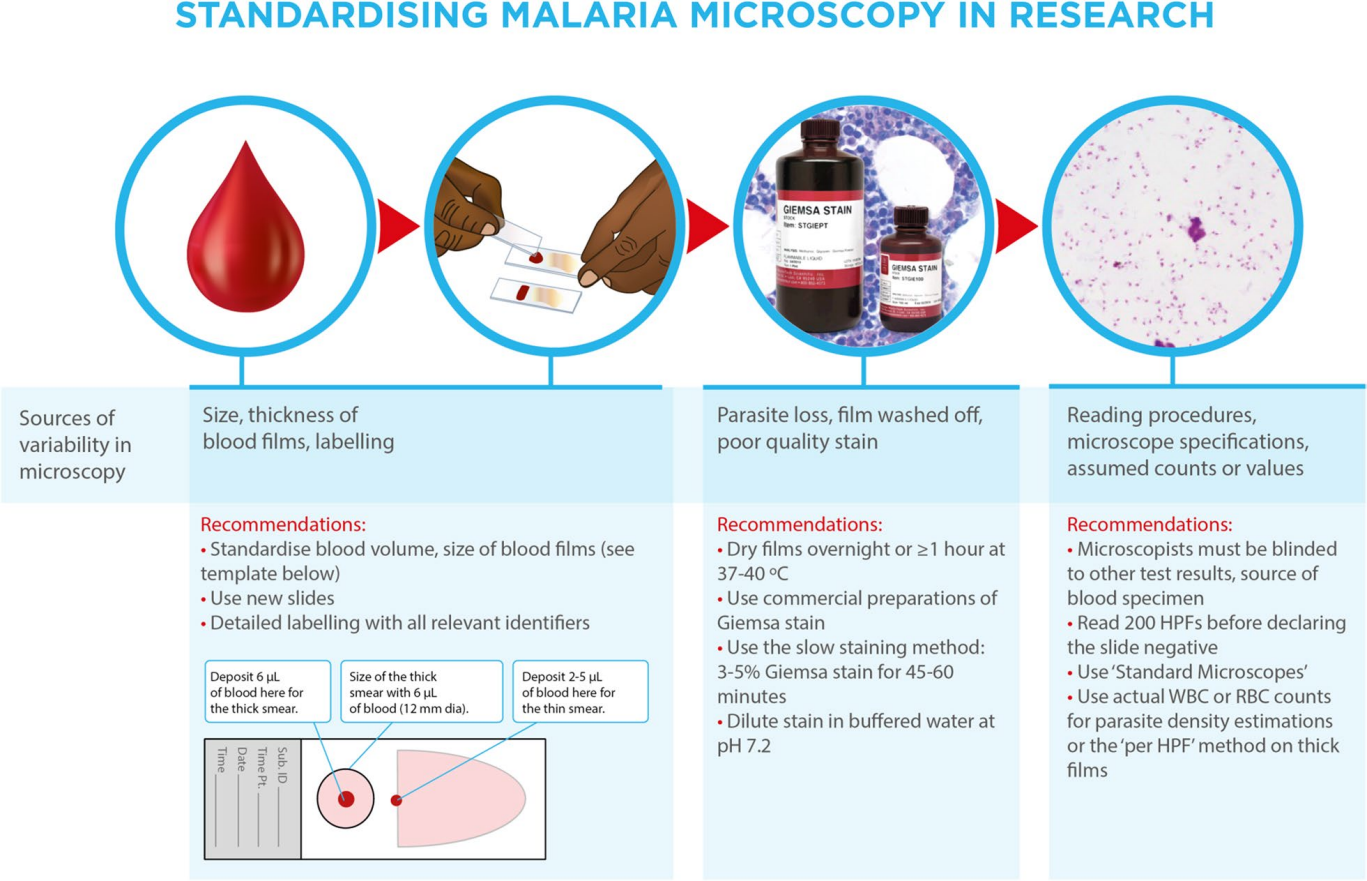
Recommended procedural adjustments to reduce variability in the detection, identification and quantification of malaria parasites by microscopy. The size of the thick film in printed versions of the template above must be verified before use

BOX 2: Recommended equipment and materials for research malaria microscopy [11] • Slide template (see Fig. 1): The template must be approximately 25 × 75 mm, i.e., same size as a slide and must have a circle of known diameter (e.g., 12 mm) situated towards one end the template such that there is adequate space for labelling and for spreading the thin film on the slide

• Micropipette to measure 5–10 µL volumes

• Giemsa stain, buffer pH 7.2—commercial preparations preferable or methods from WHO manuals or standard operating procedures.

• pH meter calibrated for measurements at pH 7.2

• Synthetic immersion oil, refractive index 1.5

• Binocular microscopes with 10 × FN 20 oculars, 100 × oil-immersion plan-achromat objective, inbuilt electric light source.

• Grid reticle (recommended if objectives are not of plan-achromat type).

BOX 3: Minimum requirements for preparing blood films and labelling slides • Labelling minimum requirement: Study + subject ID, date and time of blood collection.

• Sampling: Blood from fingerprick or venepuncture is acceptable but should be consistent within a study and stated in the protocol and study report.

• Thick films to be spread using a known volume of blood spread over fixed area, e.g., use of micropipette to measure 6 µL of blood and a template to prepare thick films of 12 mm diameter.

• Thin films to be spread using 2–5 µL of blood to obtain a thin film with a feathered edge.

• Slides must be completely dried before staining—e.g., minimum of 1 h at 37–40 °C (may need to be longer for anticoagulated blood) or overnight at ambient temperature with low humidity.

• Thin films to be fixed with absolute methanol.

### Preparing blood films

Preparing standard thick blood films can be easily accomplished by spreading a known volume of blood over a defined area on a slide as has previously been recommended for reference slides for slide banks (see Fig. 1, Box 3) [8]. The use of micropipettes and slide templates make this procedure more consistent than those depending on visual checks of the size of the blood drops used to make the films and of the size and thickness of the films, thus reducing variability in blood film size and thickness. The use of these aids offers the added advantage of making it easier to train microscopists to prepare standard thick blood films. Even with such aids however, the variation in the thickness of blood films cannot be completely eliminated, particularly at the edges of the blood film, thus requiring certain adaptations of thick film microscopy procedures as described in a later section of this paper. Further, such aids are of only limited utility in the standardization of thin blood films—blood volumes may need to be varied depending on the technique used or the viscosity of the blood, and the desired ‘feathered edge’, i.e., the monolayer of cells at the tail end of the film, can only be consistently achieved through practice.

Parasite loss is usually not seen from thin films since they are fixed with methanol before staining but measures need to be taken to enhance parasite detection and density estimations in the thick film, given reports of 30–90% parasite loss during the staining process [12, 13]. Various methods based on acetone fixation, drying of films for more than 24 h or even abrasion of the slide surface to improve adhesion of the thick film have previously been proposed to reduce or eliminate parasite loss [14]. In practice however, overnight drying at ambient temperature with low humidity or gentle warming of slides to assist drying can minimize loss. Drying for longer durations may be required when preparing films from anticoagulated blood as they are more likely to slough off during rinsing after staining. Care must be taken to avoid excessive heat (sustained exposure at > 40 °C), which may cause heat-fixing of thick films and prevent dehaemoglobinization. Heparin-anticoagulated blood is not recommended for making blood films as it may lead to a bluish background which may interfere with microscopy [15]. Anticoagulated blood samples must be spread as soon as possible, preferably within an hour of collection, to help maintain parasite morphology. It remains unclear whether there are differences in parasite detection and density between samples collected from capillary or venous blood. It is advisable to establish consistent procedures for blood collection within a single study and to report these in publications along with the other methods employed.

Finally, it is critical to ensure that slides can be unequivocally identified through correct, clear and consistent labelling to be able to link the microscopy results to study subjects and other related data without which the conclusions from the research may not be reliable. At a minimum, slides must be labelled with a study and anonymized subject ID as well as the date and time of blood sample collection. Further aids to identifying slides and detecting and resolving errors in labelling include follow-up time-points and unique codes.

### Staining

All commonly used methods for staining blood films for malaria microscopy are based on the ‘Romanowsky effect’, wherein an acidic and a basic stain are combined to produce differential colouring of the parasite nucleus and cytoplasm [16]. The stain solutions may be purely aqueous, such as Field or Jaswant-Singh-Bhattacharjee (JSB) variants, or stains may also be dissolved in methanol with glycerol to stabilize the concentrated stain solution. This modification, developed by Gustav Giemsa in 1904, has become the most commonly used standard stain for malaria microscopy and is the method recommended by the WHO [7, 10, 17]. Staining using aqueous stains can be performed in a few seconds which makes them useful for routine clinical applications especially in peripheral settings. However, stain quality can be inconsistent given that the procedure depends on dipping air-dried blood films into the staining solutions for a few seconds. These methods are probably appropriate when only a few slides are stained per day since slides need to be stained individually. However, in research laboratory settings where staining of a consistently high quality is required to assist with accurate parasite identification and where workloads tend to be heavier, Giemsa staining using commercial stain and buffer preparations is generally preferred. This helps with making secondary morphological characteristics, such as Maurer’s clefts in *P. falciparum* and Schüffner’s dots in *P. vivax* more clearly visible and allows for staining large numbers of slides simultaneously in batches at low cost per slide. The ‘slow’ staining method with 3–5% working Giemsa solution and 45–60 min of staining is recommended for slides that may need to be stored for long periods or require repeat reading [7, 8]. This is particularly relevant in the case of research studies where repeat reads are required for internal or external quality control (IQC or EQC), often with delays of weeks or months between initial and repeat reads. The ‘fast’ staining method with 10% Giemsa and 10–20 min, or even Field’s stain, can also be used in specific cases where a quick diagnosis is required, e.g. during subject screening, for symptomatic patients or when severe malaria is suspected, but a duplicate slide must also be prepared for slow staining. In all cases, slide racks must be used for staining and the use of Coplin jars or staining troughs is not recommended to obviate even the minimal possibility of transfer of parasites between slides.

### Standardizing blood film interpretation

Malaria microscopy is a procedure for the detection, identification and quantification of parasites per unit volume of blood. This implies that a standard volume of blood must be examined before a slide can be declared negative or to be able to estimate the parasite density in a given blood sample. Some early researchers used the time spent to examine blood films as a way of standardizing the volume of blood examined [18]. While this may be acceptably consistent for detecting parasites for a given microscopist, the number of fields viewed is likely to be highly variable between different microscopists and laboratories or even over time. Methods based on counting a set number of WBCs before declaring a slide negative are commonly used in clinical settings and counting parasites per set number of WBCs is perhaps the most commonly used method for parasite density estimation in research studies. This approach can reduce the effect of varying thickness of the blood film but is also dependent on the WBC count, which can vary significantly between individuals or over time. Variations in WBC counts in turn affect the volume of blood examined to detect parasites as well as estimates of parasite density if a simultaneously obtained true WBC count is not used [19]. If on the other hand the number of high power fields (HPF, 1000× magnification) is standardized, the variability in the thickness of the blood film needs to be taken into consideration, whether it is due to varying technique when preparing the thick film or due to the slight unavoidable differences in thickness between the edges and the centre of even standard thick films prepared as described above. Very often a procedure is followed wherein the presence of parasites is assessed on the thick film per a set number of HPFs and the estimation of parasite density is begun as soon as the first parasite is seen by counting parasites and WBCs. This introduces a systematic bias towards higher estimates of parasite densities, particularly in low-density infections [20]. Thin films are not suitable for parasite detection at medium or low densities as parasites can most easily be seen where the film is the thinnest, i.e., at the tail end of the thin film, where hundreds of additional fields would need to be read to match the limit of detection or analytical sensitivity of the thick blood film [21].

For research applications, it is common to read up to 200 HPFs or count up to 2000–2500 WBCs on the thick film before declaring a slide parasite-negative. This limit of detection is theoretically as low as 3–4 parasites per µL (see Table 2), though consistently achieving such a limit of detection would be rare in practice. However, it is desirable to detect parasite densities below the common limits of clinical microscopy in studies performed with human subjects to detect persisting or recurrent infections as early as possible during follow-up and also in studies assessing diagnostic tools, some of which may have a lower limit of detection than routine microscopy [5, 6]. It is clear that many asymptomatic infections occur at densities which are lower than those that can be reliably detected even by skilled microscopists [22–24]. Manual light microscopy is not aimed at detecting or excluding such infections for which other tools such as nucleic acid amplification tests are needed.

**Table 2:** Variations in counting parameters with differences in the Field Number (FN) of the ocular lens

Field number	18	20	22	26.5
Area of HPF (mm^2^)	0.0255	0.0314	0.0380	0.0552
Volume of blood per HPF (µL)	0.00135	0.00167	0.00202	0.00293
Volume of blood per 200 HPFs (µL)	0.270	0.333	0.403	0.585
Mean WBC/HPF (assuming 8000 WBC/µL)	11	13	16	23

Values calculated assuming a standard thick film made with 6 µL of blood evenly spread over a circle of 12 mm diameter [11]

Parasite density estimates can be calculated by counting the numbers of parasites per a set number of HPFs or can be based on counts of parasites and WBCs on the thick film, or parasites and RBCs on the thin film [25]. These are most accurate when the actual WBC or RBC count from a concurrently collected blood sample is used in the calculation of parasite density [26]. A minimum of 200 WBCs are usually counted on thick films where parasites are detected, increasing up to 500 WBCs in cases of low or scanty parasitaemia (< 10 parasites counted per 200 WBC) [7]. Stochastic and systematic variations in parasite density estimates can be reduced with some simple modifications of the standard procedures such as basing both parasite detection and counting on either WBC or HPF counts (as opposed to HPF counts for detection and WBC counts for density estimates) or by always counting parasites against a minimum of 500 WBCs or 40 HPF (see Table 3-A). This would increase the number of fields ‘sampled’ from the thick film for parasite counts and also allows for those fields or WBCs seen before the first parasite is detected to be taken into account in parasite density calculations (if a parasite is first detected after counting 1900 WBCs or checking 150 HPFs, the parasite count would be reported as 1 parasite per 1900 WBC or 150 HPF). It is important to note here that the limit of detection or the parasite density in parasites per µL may be significantly different between patients or even within the same patient over time if indexed to WBC counts. For example, counting 2500 WBC corresponds to examination of 0.5 µL in a subject with 5000 WBC/µL and only 0.25 µL in a subject with 10,000 WBC/µL, both of which are within the common range for WBC counts. This variability can be minimized by preparing and staining standard slides as described above and by following procedures based on HPF counts for parasite detection and density estimation [20, 25, 27]. In either case, high to very high densities (e.g. > 20 parasites per HPF or > 0.3% parasitaemia) are best assessed on the thin film (by counting parasites against 2000 RBCs; see Table 3-B) as there may be too many parasites on the thick film for accurate counts to be obtained [7]. Secondary morphological characteristics are also more clearly seen on the thin film which can aid parasite species identification even if the parasite density is too low for counting on the thin film.

**Table 3:** Impact of stochastic variation when estimating parasite density from thick or thin films with nominated parasite density. Results from simulations of counting parasites on thick films against 200 WBC and 500 WBC (assuming 8000 WBC per µL; (A)) or on a thin film per 1000 RBC and 2000 RBC (assuming 5 × 10^6^ RBC per µL; (B)). Parasite density estimates obtained from a single read by counting parasites per 500 WBC or 2000 RBC tend to be closer to the actual density. When the parasite density is estimated from two readings, fewer paired reads are discordant per Obare criteria [30]. Parasite density estimates were defined as being discordant if there is a < 10% chance of observing the two read densities if both were random samples from the theoretical probability distribution with mean equal to the average of the first and second read densities, given that at least one of the first or second reads has a density for a particular species above 200 parasites/µL

	True parasite density (parasites/µL)						
	200	250	500	1000	5000	10,000	16,000
(A) Thick film counts							
Against 200 WBC							
*Distribution of single read (n *= *1000)*							
Mean (sd)	199.8 (89.1)	250.8 (101.6)	496.7 (138.9)	983.5 (200.1)	5018.5 (565.1)	10,041.7 (904.6)	16,001.9 (1357.9)
25th and 75th percentiles	120 and 261	189 and 314	396 and 585	839 and 1121	4619 and 5397	9412 and 10,624	15,080 and 16,956
5th and 95th percentiles	76 and 355	82 and 429	272 and 731	673 and 1320	4121 and 5989	8610 and 11,604	13,822 and 18,339
*Simulation of 2 microscopy readings (n *= *10,000)*							
% with both readings ≤ 200/uL	34.3	13.9	0.02	0.0	0.0	0.0	0.0
% requiring 3rd read (Obare criteria)	12.7	15.4	15.3	15.4	23.1	28.7	36.9
Against 500 WBC							
*Distribution of single read (n *= *1000)*							
Mean (sd)	199.7 (57.0)	248.9 (64.5)	499.5 (89.2)	998.3 (133.8)	5004.5 (354.2)	9995.1 (579.5)	15,964.0 (883.0)
25th and 75th percentiles	158 and 238	204 and 287	441 and 559	910 and 1085	4756 and 5247	9607 and 10,372	15,395 and 16,556
5th and 95th percentiles	112 and 300	144 and 365	351 and 648	780 and 1213	4441 and 5605	9076 and 10,992	14,534 and 17,524
*Simulation of 2 microscopy readings (n *= *10,000)*							
% with both readings ≤ 200/µL	25.3	4.9	0.0	0.0	0.0	0.0	0.0
% requiring 3rd read (Obare criteria)	1.9	3.0	2.5	2.8	5.5	9.8	16.9

	True parasite density (parasites/µL)						
	16,000	20,000	32,000	64,000	128,000	200,000	250,000
(B) Thin film counts							
Against 1000 RBC							
*Distribution of single read (n *= *1000)*							
Mean (sd)	16,328.4 (8892.7)	19,979.2 (9541.3)	32,539.0 (12,592.1)	63,489.3 (17,600.1)	127,011.1 (23,853.1)	200,712.7 (32,038.7)	252,236.6 (33,445.7)
25th and 75th %iles	9609 and 22,386	13,915 and 24,919	23,747 and 39,969	51,450 and 75,794	110,391 and 142,273	179,076 and 221,459	229,845 and 274,556
5th and 95th %iles	4617 and 33,049	4818 and 37,912	14,020 and 53,447	34,967 and 93,502	89,799 and 168,045	147,499 and 254,466	194,879 and 306,865
*Simulation of 2 microscopy readings (n *= *10,000)*							
% requiring 3rd read (Obare criteria)	11.5	12.1	13.6	14.5	11.9	14.5	10.6
Against 2000 RBC							
*Distribution of single read (n *= *1000)*							
Mean (sd)	16,116.9 (6051.3)	20,140.1 (6869.9)	3773.0 (9055.7)	64,819.4 (12,493.7)	128,216.7 (17,857.2)	199,747.5 (22,125.0)	249,103.6 (25,238.4)
25th and 75th %iles	11,872 and 19,666	15,956 and 24,070	25,668 and 37,926	56,610 and 72,317	115,908 and 140,332	183,871 and 215,118	231,741 and 265,957
5th and 95th %iles	7076 and 26,527	9397 and 31,699	18,248 and 47,561	45,282 and 86,739	99,791 and 159,231	164,356 and 235,178	209,136 and 291,824
*Simulation of 2 microscopy readings (n *= *10,000)*							
% requiring 3rd read (Obare criteria)	2.5	3.1	4.6	3.7	3.5	3.3	3.5

Results reported from malaria microscopy need to indicate clearly whether or not parasites are detected, and if the presence of parasites is confirmed, the species and stages (e.g., asexual and sexual forms or rings and schizonts as required by the objectives of the study) of the parasites along with a parasite count against HPFs or WBCs. Depending on the requirements of the study, microscopists may choose to count parasites separately by species and stages, each against at least 40 HPFs or 500 WBCs. If parasites are seen after the first 40 HPFs or 500 WBCs, counting may be stopped and the results reported per the exact number of HPFs viewed or WBCs counted. It may be difficult to distinguish species of individual parasites on the thick film, especially at low densities, and the parasite counts in mixed infections can be combined for reporting but each detected species should be clearly indicated. Depending on the requirements of the study, gametocytes may need to be reported and counted, whereas schizonts are counted along with other asexual parasite forms (Box 4). In all cases however, parasite counts must be reported as raw data for conversion into parasite densities (see Table 4 for an example of a reporting format compliant with the Clinical Data Acquisition Standards Harmonization format from the Clinical Data Interchange Standards Consortium) [28].

**Table 4:**
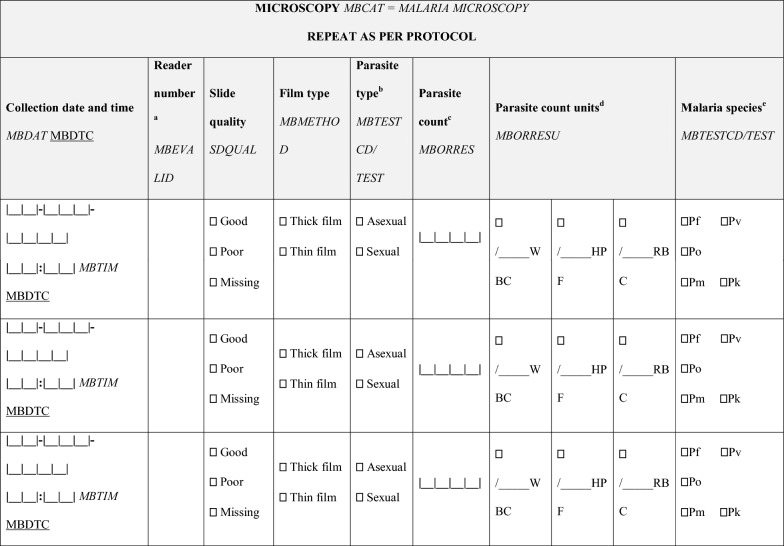
Reporting format for malaria microscopy data

Clinical Data Acquisition Standards Harmonization (CDASH) annotations are in *italics*; Standard Data Tabulation Module (SDTM) annotations are underlined

^a^For studies that require the slide to be read by more than one microscopist, include a separate row for the results from each reader

^b^Complete a separate row for asexual and sexual parasites if seen on the same slide. Adapt if additional information on the staging of asexual parasites may be required by the study protocol; e.g. rings, trophozoites, schizonts

^c^Record the actual parasite count per WBC/HPF/RBC; conversion into parasites per µL are performed separately

^d^The preferred method of calculating parasite density uses actual WBC/μL; some study protocols may assume (xxx) WBC/μL or use the ‘HPF’ method

^e^In cases of mixed infections, all infecting species must be reported; however, the asexual and/or sexual parasite count need not be reported separately for each species unless specifically required in the study protocol. If species are reported separately, counts for each species must be entered on separate lines. Asexual and sexual stages from the same slide/parasite species also must be entered on separate lines

BOX 4: Standard parameters for blood film examination and parasite counting • Slides must be examined at 1000× magnification using immersion oil and adapted objective lenses.

• A minimum of 200 HPFs must be examined or 2500 WBCs counted on the thick film before declaring a slide negative.

• Clearly defined criteria must be consistently applied to thick and thin films to count parasites for density estimation (e.g., if parasite density is > 20 parasites per HPF or > 2 parasites per WBC, parasite density estimation must be performed using the thin film).

• Parasites must be counted in a minimum of 40 HPFs or against 500 WBCs in the thick film or against 2000 RBCs in the thin film.

• Results must be reported as raw figures, i.e. species of parasites, the number of asexual or sexual forms of the parasite over the number of WBCs or fields counted.

• The most recently measured actual WBC or RBC counts for the study subject must be used for parasite density estimation.

### Quality assurance

Quality control (QC), which in the context of microscopy usually means re-checking of slides already examined by a microscopist, is a critical component of any quality management system since it provides feedback on the quality of outcomes obtained from routine processes implemented under everyday conditions. For malaria microscopy, QC must also include assessments of the quality of blood films and staining as poor quality slides can directly affect the ability of the microscopist to detect, identify and count parasites. Deviations from pre-defined tolerances can help to identify needs for adjustments or refinements to established procedures and corrections to individual outcomes, while also indicating if more rigorous QC protocols are required. In malaria microscopy, such tolerance limits have been defined for detection, identification and estimation of parasite density and are described below. These limits have also been defined in Box 5 along with recommendations on numbers or proportions of slides to be rechecked for IQC and EQC. It stands to reason that a higher proportion of slides would need to be rechecked if the initial microscopy is performed by inexperienced or uncertified microscopists, but a certain minimum must be maintained to monitor the quality of microscopy. QC protocols must be carefully designed to address the particular needs of the study while adhering to the minimum standards defined below and must take into account blood film and staining quality. The results from such quality checks can be used to monitor the performance of individual microscopists by comparing their results with the reported consensus results and, depending on their performance, to adjust the proportions of slides to be reread. External QC performed by certified assessors provides an additional check of conformity to the standards required for research malaria microscopy and can detect systematic errors or those which are made by all microscopists at a site.

Definitions of acceptable tolerance in the detection and identification of parasites are straightforward—if results from two independent reads on the same slide are not in agreement with respect to the presence of parasites or their species then a tiebreaker read is needed. Defining the tolerance for variations in parasite density requires more sophisticated methods. The implementation of such QC criteria or protocols can be facilitated and made systematic by using computing aids which standardize the thresholds for cross-checking by a third microscopist and acceptance criteria for managing outlying results across studies, producing more predictable variances when comparing paired reads for concordance [29–31]. The Obare Method Calculator, for example, assesses the probability of observing the two estimated densities (given at least one of the two has a density > 200 parasites/µL) if both were random samples from the theoretical distribution of densities with mean equal to the mean of the two estimates. If this probability is < 10%, the paired reads are identified as being discordant and a third read is required. Other methods, based on calculations of the difference in the square roots of the reads or on proportional differences of reads from their mean have also been previously described and could be adequate to identify slides requiring third reads.

BOX 5: Guidelines for internal and external quality control (IQC and EQC) of malaria microscopy • All microscopists performing QC reads must be blinded to the slide details as well as the initial results.

• QC checks performed prior to the initial examination of the slide and during re-checking must include assessments of slide quality per standard criteria. The slide(s) must be rejected if any issues that could affect parasite detection and/or counting are identified (e.g., excessive artefacts or stain precipitate, non-standard stain or pH interfering with parasite detection and/or identification, fixed or patchy thick films).

• IQC: minimum 20% of all study slides, randomly selected, re-read for IQC; in case the study microscopists have been certified at Competency Levels 3 or 4, or are uncertified, up to 100% double-reads may be required depending on QC results.

• Results from paired reads from the same slide are considered discrepant and require a third tie breaker read if:

• Only one microscopist detects parasites;

• The species of the parasites reported by the microscopists is (are) different;

• The difference in parasite density for asexual parasites is greater than a pre-defined limit (e.g. per the Obare Method Calculator, ‘square mean root count’ method [29], or a  % difference from the mean of the two reads). An exception is when both estimates are ≤ 200/µL, in which case both parasite density estimates are considered to be within accepted ranges of variability as long as both detect parasites. Sexual forms and schizonts can be reported separately but, depending on the study’s needs, may or may not need to be considered in assessments of discordance.

• EQC: 5 positive and 5 negative slides per microscopist per month or ≥ 5% of all slides if > 200 slides read per month, randomly selected, to be re-read by external assessors with certified Level 1 Competency.

### Recording and reporting microscopy results

To enable useful interpretation of results and comparisons with other studies, standardized reporting of the results and methods used for microscopy is essential, whether the microscopy is the primary subject of the study or a standard against which other assays are compared. Standardized recording of microscopy results may be easily implemented by using case record forms compliant with the Clinical Data Acquisition Standards Harmonization [28]. Further, reports involving microscopy should clarify the competence of the microscopists, technical specifications of the microscope and the methods and reagents used, all of which contribute to accuracy of slide interpretation (Box 6). A description of the quality assurance processes in place must also be reported alongside the QC results which help to demonstrate the reliability of the results presented.

BOX 6: Suggested minimum requirements for reporting malaria microscopy procedures and results in clinical research publications • Blood sample collection method (fingerprick or venepuncture).

• Source of Giemsa stain stock solution.

• Microscope specifications and/or brand, model.

• Number of fields examined before declaring slide negative.

• Parasite density estimation method (‘WBC’ or ‘HPF’); WBC, RBC counts used in calculations (actual or assumed counts).

• QC protocols (competency testing done by microscopists if any, proportion of slides double-read, criteria for tie-breaker reads, proportion of slides rechecked for external quality control, competency level of reference microscopist).

• QC results (as compared to reference microscopy—Se, Sp, *kappa* for species, proportion of slides with discordant parasite density as detected by external rechecking).

## Discussion

The Research Malaria Microscopy Standards (ReMMS) presented here, complemented by the ReMMS Manual and the Obare Calculator research microscopy standardization tool, are an attempt to make a set of standards and procedures freely available to the malaria research community, aimed at increasing the consistency and reliability of malaria microscopy performed for research studies (see Box 6) [11, 30, 32]. This is a vital and necessary step towards harmonizing the methods used in malaria studies which will allow data obtained over time and/or across diverse contexts to be compared directly, thus multiplying the impact of and confidence in studies assessing drug or vaccine efficacy or the performance of new diagnostic tools. While certain points in these standards and procedures are highlighted as being critical to maintaining consistency, it is hoped that there is enough flexibility to accommodate the particularities of diverse study types and that they will be widely adopted. They provide the core of a quality management system and can be used to design programmes for training, proficiency testing and quality assurance of research malaria microscopy.

Many research laboratories have already implemented quality management systems for microscopy or for the laboratory as a whole. However, a framework, such as the one described in the WHO Malaria Microscopy Quality Assurance manual for National Malaria Control Programmes, is lacking for microscopy in research. As a result, high-quality training and standard proficiency testing is hard to find outside certain reference centres [33]. While this is being addressed, it is hoped that researchers will find it worthwhile to review and update their existing quality management protocols to match the standards described here using currently available resources (e.g., WHO Malaria Slide Bank hosted by the Research Institute for Tropical Medicine [The Philippines], Malaria Research and Reference Reagent Resource Center [USA], slide exchanges with collaborating or partner laboratories) for testing the proficiency of their microscopists. It would be hard to over-emphasize the importance of the high level of motivation and commitment needed from microscopists and their management to attain and maintain these standards. This process would be facilitated by ensuring the availability of training on the techniques and by recognizing the importance of workload management, feedback on performance and support for improvement where needed. These are all factors that should be part of a well-functioning quality management system and will need to be in place if laboratories are to attain the goal of certification of microscopists and accreditation of the microscopy laboratory. Without sufficient effort to ensure high standards and repeatability of microscopy results within field study design, the effort and resources employed, and the support of study subjects is greatly devalued.

Microscopy on Giemsa-stained blood films to detect, identify and quantitate malaria parasites is a technique which is already more than a century old and may be replaced in the future by methods based on polymerase chain reaction (PCR) or machine learning for micrographic image analysis for research and clinical applications [34, 35]. PCR-based methods have the advantage of being less dependent on human interventions for interpreting and reporting of results but are typically more demanding in terms of human and material resources, training and quality assurance. They may require significant investment into robust logistics to transport samples to a central laboratory or for equipping peripheral laboratories with PCR equipment, or both, and have their own challenges with respect to standardization between laboratories [36]. Turn-around times for results may be higher due to requirements for batch processing to conserve reagents making it difficult to use PCR-based testing for screening symptomatic patients or for follow-up. Further, quantification of parasite density is complicated by multinucleate forms of the parasite (mostly for non-falciparum species) and by the requirement for separate RT-PCRs required for gametocyte detection. Overall, a wealth of information, including that on other concurrent blood-borne parasites or haematological parameters, can be obtained from correctly performed microscopy. Such analyses can also be performed by PCR-based or other technologies but at a much higher complexity and cost.

## Conclusion

The standards described here (Fig. 1) and the accompanying manual were developed to guide a move towards common standards for undertaking and reporting research microscopy for malaria parasite detection, identification and quantification. These documents are based on agreed quality assurance standards for research malaria microscopy defined by participants in an informal consultation convened by the Special Programme for Research and Training in Tropical Diseases (TDR). The participants jointly recognized a need to standardize research microscopy to improve the quality of clinical and diagnostic trials, enable comparisons of outcomes between clinical trials, and provide clarity in publication. It is hoped that this summary, together with the Manual it describes and the accompanying Obare Calculator, will form a solid basis for the wider adoption of standardized reference microscopy protocols for malaria research.

## Data Availability

Not applicable.

## Abbreviations

µL: Microlitre
EQC: External Quality Control
FN: Field number
HPF: High-powered field (1000× magnification)
IQC: Internal Quality Control
O/n: Overnight
PCR: Polymerase chain reaction
QC: Quality control
RBC: Red blood cells
ReMMS: Research Malaria Microscopy Standards
RT-PCR: Reverse transcriptase-polymerase chain reaction
TDR: Special Programme for Research and Training in Tropical Diseases
WBC: White Blood Cell
WHO: World Health Organization
